# The Role of Servant Leadership in Work Engagement Among Healthcare Professionals

**DOI:** 10.3390/healthcare13202565

**Published:** 2025-10-12

**Authors:** Vesna Malićanin, Aleksandar Čivović, Ana Aničić, Marijana Bugarčić, Marko Slavković

**Affiliations:** 1 Health Centre Dr Nikola Dzamic, 21 Kraljevacka Street, 36210 Vrnjacka Banja, Serbia; vesna.malicanin@dzvbanja.org.rs; 2 Health Center Gornji Milanovac, Vojvode Milana 37, 32300 Gornji Milanovac, Serbia; aleksandar.civovic@dzgm.rs; 3 Batočina Health Center Gornji Milanovac, Kneza Miloša Obrenovića Street 1, 34227 Batočina, Serbia; ana.anicic@dzbatocina.rs; 4 Faculty of Economics, University of Kragujevac, Liceja Kneževine Srbije 3, 3400 Kragujevac, Serbia; marijana.bugarcic@ef.kg.ac.rs

**Keywords:** servant leadership, leadership style, work engagement, quiet quitting

## Abstract

Background/Objectives: Healthcare organizations worldwide face challenges in retaining talented employees, with the phenomenon of quiet quitting increasingly recognized as a contemporary issue. Rather than leaving their jobs, employees remain at work but exert minimal effort and exhibit reduced engagement, which can ultimately undermine the performance of healthcare organizations. The aim of this research was to examine the impact of servant leadership on work engagement within healthcare organizations, to determine whether this leadership style can help mitigate the effects of quiet quitting. Methods: The study employed a quantitative approach, utilizing validated instruments to measure servant leadership and work engagement. A cross-sectional study design was utilized, employing a convenience sampling method. A total of 362 valid surveys were collected from healthcare professionals in Serbia participating in the study from January to March 2025. The partial least squares structural equation modeling (PLS-SEM) method was used to examine the relationship between servant leadership and work engagement among healthcare professionals. Results: The results indicate that servant leadership has a positive and statistically significant impact on all dimensions of engagement: vigor, dedication, and absorption. Conclusions: Based on these findings, it is concluded that servant leadership can serve as an effective strategy for enhancing work engagement and reducing negative employee behaviors, such as quiet quitting, which may, in turn, improve organizational efficiency in the healthcare industry.

## 1. Introduction

While healthcare workers traditionally left their jobs in search of better opportunities, more recently, the phenomenon of quiet quitting has emerged. This refers to employees remaining in their positions but significantly reducing their efforts, completing only the assigned tasks and not putting in extra energy to achieve additional performance [1]. These employees meet the minimum criteria for their tasks, avoid coming to work earlier or staying later, and refrain from exceeding established standards [2]. This phenomenon became particularly pronounced during the COVID-19 pandemic, primarily driven by employees’ desire to achieve a better work–life balance [3]. As a result, employees invest only the effort necessary to avoid dismissal [4]. Such employees continue to work with low motivation and energy, often accompanied by high frustration, which over time can lead to a decline in performance or other issues [5].

Quiet quitting can be attributed to various factors, including poor interpersonal relationships, burnout, decreased job satisfaction, and unfavorable working conditions [4]. Moisoglou [2] particularly highlights the role of leadership style in organizations as a potential strategy for reducing and eliminating quiet quitting. The role of a leader goes beyond merely leading; it also involves serving employees within a healthcare organization [6]. Such a leader must prioritize the needs of employees [7], especially considering the challenges of finding and retaining talented staff. Patients seek quality service, and the management of healthcare organizations values efficiency, which cannot be achieved if the leadership style does not align with the needs of healthcare workers and the contextual factors [8]. A servant leader can offer employees opportunities for growth and development while addressing their other, primarily intrinsic needs, thereby fostering a positive impact on work engagement [9,10]. However, despite numerous studies, the concept of work engagement remains insufficiently explored within healthcare organizations [11]. As noted by Szilvassy and Širok [12], this concept has been especially under-researched in primary healthcare, despite the crucial role of employee engagement in ensuring quality care.

As a social phenomenon, leadership involves the process of engaging resources, capacities, and leading employees toward achieving the organization’s goals, with its effectiveness depending on various contingent factors [13,14]. An effective leader acknowledges these factors when creating and communicating a vision for employees, encouraging and utilizing their creativity and innovation, especially within healthcare organizations [13,15]. In the context of healthcare organizations, servant leadership stands out as a unique approach, characterized by moral principles and the leader’s focus on achieving organizational goals by prioritizing the needs of employees [16]. As the name of this leadership style suggests, the leader’s role is to serve employees, concentrating on maximizing their potential to meet the needs of the organization. A servant leader does not aim to provide specific benefits to employees but seeks to enhance their well-being, motivation, and efficiency [17]. Other key attributes of servant leadership include the desire to promote the well-being of followers, trustworthiness, integrity, and the recognition of others’ potential [14].

The effectiveness of servant leadership in healthcare organizations is primarily reflected in its positive impact on the attitudes and behaviors of healthcare workers, which in turn influences the quality of healthcare and patient satisfaction [8]. Additionally, relational dynamics with followers should not be overlooked, as the leader, in exchange for their behavior, provides development opportunities, collaboration, and a role model for employees to follow [18]. As noted by Bavik [19], honesty and integrity are central components of servant leadership, but ethics, openness, altruism, empowerment, compassion, listening, empathy, and others are also emphasized [20,21,22]. Therefore, a servant leader fosters employees’ self-confidence and emotional stability, which ultimately impacts the overall performance of the organization [14,17,22]. Servant leadership is characterized by empowering followers to make decisions, holding them accountable for those decisions, stepping back to prioritize the needs of others, demonstrating humility by valuing employees’ knowledge, authenticity, the courage to make new decisions, understanding and accepting interpersonal relationships, and stewardship, that is putting the needs of the majority or the organization above personal interests [23]. Thus, it can be said that servant leadership has both a strategic and an operational aspect, with the former involving the ability to create and communicate a high-purpose vision and behavioral model, while the latter focuses on caring for others and monitoring work outcomes [18].

Work engagement represents a unique sense of satisfaction and a positive mental state resulting from the work being performed [24,25,26]. Although employee engagement is important in any job, it is especially crucial in healthcare organizations due to the psychological and emotional nature of services provided to patients, which require high levels of intrinsic motivation [27]. Work engagement refers to the physical, emotional, and cognitive connection an employee has with their tasks [28]. It indicates the degree of commitment and the level of energy and effort an employee invests in order to accomplish a task [29]. Often considered an indicator of intrinsic motivation, work engagement is expressed through three components: vigor, dedication, and absorption [25]. Vigor refers to the energy and effort an employee applies to their work, and their perseverance in completing tasks. Dedication involves the emotional commitment, enthusiasm, and drive to complete tasks. Absorption refers to the state of being fully immersed and focused on tasks, indicating high levels of concentration and engagement [26,27,28,29].

Work engagement is of strategic importance for healthcare organizations aimed at improving the quality of healthcare. The engagement of healthcare workers significantly impacts patient safety, both emotional and psychological, and ultimately affects the performance of the entire organization [30]. Today, healthcare organizations generally implement numerous policies and procedures to enhance service quality [31]. The quality of healthcare services primarily depends on the quality of employees’ work, which in turn is determined by various factors influencing employee behavior and job satisfaction [32]. As noted by Perić et al. [33] and Wee and Lai [34], the growth in job satisfaction is positively correlated with improvements in service quality, including healthcare services. Therefore, leaders and managers of healthcare organizations are expected to create working conditions that promote the work engagement of healthcare workers [35]. Coetzer et al. [18], Bavik [19], and Eva et al. [36] report a positive influence of servant leadership on work engagement in various settings. A focus on the needs of subordinates fosters better social relationships with the leader [10]. In healthcare organizations, such relationships can facilitate better knowledge sharing, especially given the diverse human capital within healthcare [37]. A servant leader provides employees with opportunities for development, the acquisition of new knowledge, and its application in their work, fostering positive intrinsic motivation, one of the antecedents of work engagement [38]. Therefore, a servant leader motivates and ensures greater employee dedication to their work [39]. In such cases, work engagement increases due to the higher level of innovation and creativity from employees who have the autonomy to apply their knowledge in practice [22,40].

Ozturk et al. [40] emphasize that the influence of servant leadership on work engagement has a stronger impact on job satisfaction, while Kaya and Karatepe [41] argue that servant leadership exerts a more significant influence on work engagement compared to other leadership styles. Given the significance of this impact, it can be assumed that the implementation of servant leadership could be an effective strategy for reducing quiet quitting. Leadership is often identified as one of the strategies that should mitigate the effects of this phenomenon, and thus, appropriate leadership support can help reduce or eliminate quiet quitting [2,42]. By enhancing work engagement, servant leaders decrease the likelihood of employees leaving the organization [16], thereby preserving valuable and rare talents.

To address patients’ desire for the highest quality care, and which needs to cost less, healthcare organizations should manage changes focusing on the strength of the team, developing trust, and serving the needs of patients. Trastek et al. [43] emphasized the importance of servant leadership in the long-term stability of healthcare organizations, arguing that it is essential for the treatment of patients and the collaboration of staff. Servant leadership in healthcare centers leaders on serving patients and staff first, cultivating trust, psychological safety, and a people-oriented culture that empowers and develops healthcare professionals [44]. This approach is linked to stronger teamwork and provider–patient relationships, higher employee satisfaction and commitment, which can result in improvements in patient satisfaction. A study in Saudi Arabian healthcare organizations revealed a positive relationship between servant leadership and leadership effectiveness [45], indicating that this leadership style enhances the work-related outcomes of leaders, in addition to benefiting their followers. Systematic review on the servant leadership in the healthcare settings revealed that this leadership style is consistently related to positive individual and organizational outcomes, and identifies social exchange theory as the dominant explanatory mechanism [16]. Social exchange theory frames workplace relationships as ongoing exchanges of socio-emotional and material resources governed by reciprocity, trust, and obligation. Servant leadership well fits this logic by prioritizing follower growth and needs, thereby initiating high-quality exchanges that elicit reciprocal commitment and citizenship. Integrative reviews position servant leadership as a values-based style whose effects are frequently interpreted through social exchange theory mechanisms [46,47]. By investing in followers, servant leadership acts as a catalyst for social exchange, fostering trust, obligation, and identification that extend from individual relationships to teams, resulting in citizenship, engagement, and performance [36]. Accordingly, healthcare organizations should prioritize the development of servant leadership, which, through social exchange mechanisms of trust, reciprocity, and obligation, enhances follower citizenship and engagement.

Previous studies carried out in the healthcare industry have confirmed the relationship between servant leadership and work-related outcomes, such as job performance [8] or job satisfaction [48]. Also, the association between servant leadership and work engagement has been established in a study involving service sector employees [9], as well as in research conducted with high school and vocational high school teachers [10]. On the other hand, research on the relationship between servant leadership and work engagement in the healthcare industry still remains limited. A more recent study in the United States involving 57 participants investigated the correlation between servant leadership and work engagement in healthcare settings [49]. The limited sample size in that study, as well as the absence of similar studies exploring the impact of servant leadership on work engagement in the healthcare industry, especially in developing countries, poses a clear research gap that our study tries to address. Additionally, the results of the study are related to the phenomenon of quiet quitting, which appears as a global challenge in healthcare settings. This is the first study to systematically examine how servant leadership affects work engagement in the Serbian healthcare industry, aiming to determine if it can create the potential to reduce quiet quitting.

In this context, the primary question that our study aims to explore, including healthcare professionals as participants, is as follows:

RQ1: What is the effect of servant leadership on healthcare professionals’ work engagement?

To the authors’ knowledge, the relationship between servant leadership and vigor, dedication, and absorption as components of work engagement has not been previously investigated. Consequently, our work presents a secondary research question to explore these effects:

RQ2: Does servant leadership affect vigor, dedication, and absorption and thereby create the potential that each individual relationship can contribute to reducing quiet quitting?

**Figure 1:**
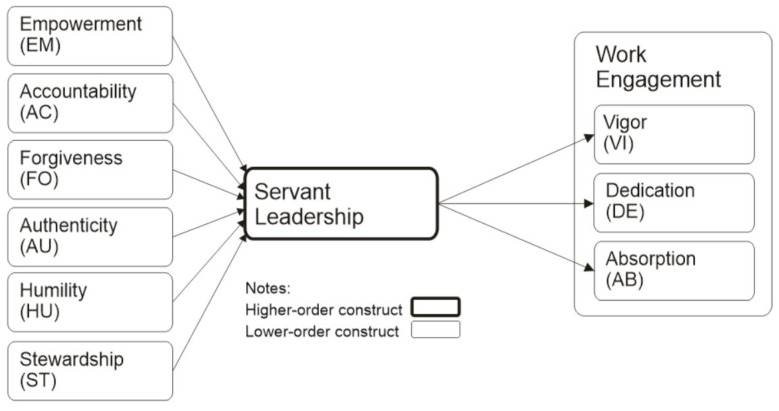
Research model.

A research model was developed utilizing the details outlined above, as illustrated in Figure 1.

## 2. Materials and Methods

### 2.1. Sampling Methods and Procedure

To report the research questions, an empirical study was conducted on a sample of healthcare professionals in the Republic of Serbia. The cross-sectional study design involved healthcare professionals employed in healthcare organizations of all types in Central Serbia, including Belgrade, as the capital, excluding administrative staff. The sample procedure utilized the convenience sampling method, with each participating healthcare professional requested to refer others who met the study’s criteria, thus facilitating the recruitment of additional participants via snowball referrals. The questionnaire, composed in Serbian, was disseminated electronically from January to March 2025. Even though convenience sampling limits the extent to which the results may be generalized statistically, sample bias was reduced by recruiting across a diverse range of healthcare organizations on several weekdays, which in turn increased the demographic diversity of the sample.

To address ethical considerations, informed written consent was obtained on the first page of the electronic questionnaire. Before participation, respondents were informed of the academic purpose of the study and assured anonymity, which was further supported by the online mode of data collection. Participants were also advised that their demographic information would be used exclusively for statistical analysis and would be strictly limited to gender, age, and years within healthcare organizations. Participants were assured that the data would be protected from access by any third party. The questionnaire excluded items on highly sensitive personal data to further protect the identity of respondents and to avoid receiving socially desirable answers. Participants were assured that all data would remain confidential and inaccessible to third parties. Participation was entirely voluntary, and respondents could complete the survey at their convenience. The study was conducted in accordance with the Declaration of Helsinki and approved by the Council of Interdisciplinary and Multidisciplinary Studies, University of Kragujevac (protocol number IV-07-384/4 from 20 May 2024).

Before starting the sampling procedure, the minimum required number of participants was determined using the inverse square root method [50]. For the maximum number of arrows pointing at a construct projected by the design of the research model at 10, the estimated sample size was 189 respondents. Three months into the research, the sampling procedure was halted after distributing 500 questionnaires. The response rate was 72.4%, resulting in a sample of 362 respondents, whose characteristics are presented in Table 1.

In the sample, a notable participation of female respondents (87.3%) was observed, with 49.2% of participants being under the age of 40, and the majority having an average length of service in a healthcare organization exceeding 20 years (34.8%). While the sample’s predominant female respondents may be cause for concern, the overall population of healthcare professionals in Serbia is comparable to that of the sample. It consists of 68% females and 32% males [51], showing a significantly higher presence of female healthcare professionals than males, which makes the sample suitable for further statistical analysis.

### 2.2. Survey Instrument and Measurement

Two variables were constructed in the research process: the independent variable, which refers to servant leadership, and the dependent variable, which refers to work engagement. Servant leadership was measured using the Servant Leadership Survey [23], which examines servant leadership through eight components: empowerment, accountability, standing back, humility, authenticity, courage, forgiveness, and stewardship. This measurement scale has already been employed in healthcare settings [52] and was hence considered appropriate for the purpose of this research. The items in the Servant Leadership Survey were initially composed in English. The scales were translated into Serbian, and their measurements were adapted to align with the Serbian context. Pretests were conducted with a sample of 30 respondents to establish the instrument’s validity and provide feedback on potential alternative wording if needed. The dependent variable, work engagement, was measured using the Utrecht Work Engagement Scale (UWES), specifically its longer version [53], which was verified in Serbian [54]. Work engagement was operationalized through the components of vigor, dedication, and absorption. The UWES, in its long form, was chosen to comprehensively assess work engagement, encompassing vigor, dedication, and absorption through multiple indicators for each facet, thus improving content validity and measurement accuracy. The longer form also supports more robust confirmatory factor analysis and structural equation modeling. All respondents rated their attitudes on a five-point scale, where 1 represented “complete disagreement” and 5 represented “complete agreement” with the stated statement. In addition to the aforementioned sections, which were grouped into two parts, the questionnaire also included a third part designed to collect the socio-demographic characteristics of the respondents.

In this research, the partial least squares approach to structural equation modeling (PLS-SEM) was employed within a formative higher-order model to identify the statistical significance of the relationships between the observed variables and to provide answers to the defined research questions. This research approach is justified by two key arguments. First, as noted by Hair et al. [55], the use of PLS-SEM is appropriate when the data do not follow a normal distribution. Second, PLS-SEM is frequently applied in studies related to marketing and human resource management [56,57]. Given that this study involves two variables—servant leadership and work engagement, both of which are closely linked to human resource management—this analytical approach is particularly suitable. Furthermore, the primary objective of the study is to predict and explain variance in the key outcomes, and considering the complexity of our model, which includes a higher-order latent variable with numerous indicators, the PLS-SEM approach is selected. It reports well reliability and validity for the measurement model and focuses on predictive criteria. Statistical data processing was conducted using the SPSS V.23 and SmartPLS v4.0 statistical software packages.

## 3. Data Analysis

Results for the evaluation of model reliability and validity appear in Table 2. The results confirming the construct were obtained using confirmatory factor analysis [57]. As shown in Table 2, average variance extracted (AVE) values exceed 0.50 for all cases, supporting convergent validity and implying that more than 50% of item variance is captured by their respective constructs [58,59]. The AVE values in Table 2 range from 0.764 to 0.917. Additionally, the composite reliability, which should exceed 0.7 [60], is met for all indicators in Table 2.

However, two constructs of servant leadership did not achieve the required validity: Standing back and Courage, and therefore were eliminated from further calculations. Consistent with best practice in variance-based SEM, we re-specified the higher-order servant leadership construct using the six validated first-order dimensions (empowerment, accountability, humility, authenticity, forgiveness, stewardship). This decision preserves psychometric adequacy while acknowledging a narrower content domain. Thematically, the misfit of Standing back and Courage may reflect cultural and organizational context. Serbian healthcare organizations are relatively formal hierarchy with a risk-averse climate and therefore can lack compatibility with leaders remaining in the background and giving credit to others, as well as taking principled stands and challenging the status quo, as essential characteristics of Standing back and Courage. Because the factor loading was less than 0.7, the reliability test did not satisfy two statements: (1) “My manager gives me the authority to make decisions that make work easier for me” (construct: Empowerment) and (2) “My manager emphasizes the societal responsibility of our work” (construct: Stewardship), and consequently they were eliminated. Collinearity statistics, measured by the variance inflation factor (VIF), indicate the absence of multicollinearity, as all items achieved values lower than 5, which is the upper limit [61].

The Fornell–Larcker criterion indicated that the Empowerment and Humility facets of servant leadership are not sufficiently distinct, suggesting some conceptual and empirical overlap in our context (Table 3). Substantively, this implies that behaviors reflecting giving autonomy and resources (Empowerment) and leader modesty, as well as other-orientation (Humility), are perceived as a single cluster of “empowering humility” by respondents. This result is plausibly due to specific cultural and organizational norms in Serbian healthcare settings, or it may appear due to specific circumstances in society. Furthermore, the specific context of developing countries can make a difference compared to previous studies that blur facet boundaries.

Multiple diagnostics were used to assess common method variance (CMV). Harman’s unrotated single-factor test indicated 54.14% variance for the first component. However, permutation-based parallel analysis on Spearman correlations showed that the first two eigenvalues (18.41, 2.58) exceeded the 95th-percentile null thresholds (1.70, 1.60), contradicting a dominant single-factor structure. Further, a trait-controlled residual principal component analysis (items residualized on their intended constructs) yielded a first residual component of only 7.67% variance, indicating that any remaining CMV is modest and unlikely to account for the observed relationships.

Despite expectations that all first-order servant leadership constructs contained in the original questionnaire would be validated, the results showed that the higher-order servant leadership construct contained six, instead of the original eight constructs. Excluding two first-order constructs should not undermine testing the relationship between servant leadership and work engagement link, but may improve the estimation accuracy while the six retained facets achieve satisfactory reliability and validity.

## 4. Results

Predictive relevance was assessed by calculating Stone–Geisser’s Q^2^ (cross-validated redundancy index) for the dependent variable (Table 4). The Stone–Geisser Q^2^ value for Absorption was determined to be 0.369; for Dedication, it was 0.332; and for Vigor, it was 0.378. The indicator’s positive value supports the adequacy of the structural model [62,63].

The coefficient of determination (R^2^) shows that 37.9% of Absorption is described by the model, while this value for Dedication is 33.2%, and for Vigor, it is 37.8%, indicating good explanatory power. The observed f^2^ values for Absorption (0.611), Dedication (0.518), and Vigor (0.624) all exceed the “large” threshold, indicating that servant leadership makes a substantial incremental contribution to explaining each engagement dimension and—together with positive Q^2^ values—supports strong predictive relevance. Practically, these magnitudes imply that changes in servant leadership translate into meaningful gains in vigor, dedication, and absorption. The standardized root mean square residual (SRMR) achieves a value lower than the defined 0.08 [64], which suggests a good model fit. The value of the GOF test falls within the optimal range (from 0 to 1). The results of the direct effects test are shown in Table 5. The PLS-SEM bootstrapping procedure was used to test the statistical significance of the relationships between the constructs included in the research, which meet the criteria of reliability and validity. Path coefficients and two-sided adjusted 95 percent confidence intervals (CIs) were calculated for each of the reported relationships.

**Figure 2:**
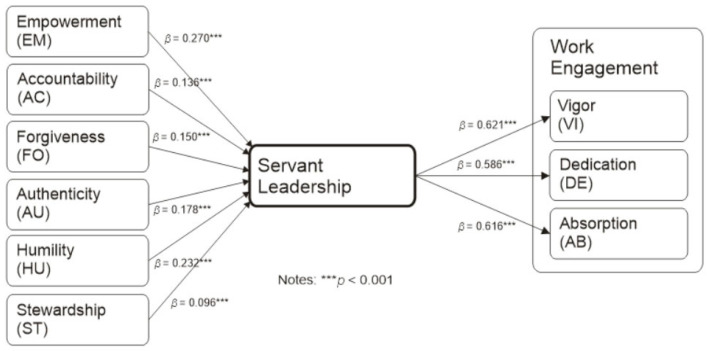
PLS-SEM path modeling. *** *p* ˂ 0.001.

The results show that the constructs constituting servant leadership have a statistically significant and positive influence in all cases (Figure 2). Specifically, Empowerment (β = 0.270, *p* < 0.001), Accountability (β = 0.136, *p* < 0.001), Forgiveness (β = 0.150, *p* < 0.001), Authenticity (β = 0.178, *p* < 0.001), Humility (β = 0.232, *p* < 0.001), and Stewardship (β = 0.096, *p* < 0.001) all demonstrate positive effects. Looking at the influence on the dependent variable, servant leadership has a statistically significant and positive effect on Vigor (β = 0.621, *p* < 0.001), Dedication (β = 0.586, *p* < 0.001), and Absorption (β = 0.616, *p* < 0.001). Specifically, a one-point increase in servant leadership results in a 0.616 increase in Vigor, a 0.586 increase in Dedication, and a 0.616 increase in Absorption.

The robustness test was conducted using the SmartPLS MGA (multi-group analysis) option to ascertain the consistency of the results across various subgroups. Before conducting the MGA, the categorical variable “Years within healthcare organization” was recoded so that instead of five values (<5; 5–10; 11–15; 16–20; >20), it contains three values (<10; 10–20; >20), compelling a more effective distinction.

A multi-group analysis (MGA) showed invariance in all relationships between servant leadership and the dimensions of work engagement (vigor, dedication, and absorption) across different subgroups (Table 6). Within different groups, the *p*-values were statistically significant (*p* < 0.001), confirming that the effect of servant leadership exists across all subgroups. However, the β and t values revealed differences in the strength of this influence. In relation to age and years within the healthcare organization, the differences in β values were moderate—servant leadership had the strongest impact on younger employees (<40) and on those with 10–20 years of service. Regarding gender, the differences in the β coefficients were discrete, but higher t values among women indicated a slightly stronger effect of servant leadership on their engagement, particularly on the vigor, dedication, and absorption. Moderate differences in β were observed within age. Servant leadership had the strongest effect on younger employees (<40) across all three dimensions of engagement, most notably on vigor (β = 0.729) and absorption (β = 0.698), while dedication was also high (β = 0.688). For employees aged 41–50, the effect remained present but somewhat weaker, whereas in the oldest group (>51), it was the weakest, particularly with regard to dedication (β = 0.343). This indicates that the influence of servant leadership on work engagement gradually decreases with age. Concerning years within a healthcare organization, the strongest effect of servant leadership across all dimensions was recorded among employees with 10–20 years of service (VI—β = 0.760; DE—β = 0.724; AB—β = 0.751). For those with less than 10 years of service, the effect was moderate, while among employees with more than 20 years, it was the weakest, especially in relation to dedication (β = 0.403). This suggests that servant leadership has the greatest influence during the early stage of professional development, while its impact is more limited among the longest-serving employees, likely due to greater independence and established work patterns.

## 5. Discussion

The primary objective of this research was to explore the impact of servant leadership on work engagement, particularly in relation to reducing or eliminating the phenomenon of quiet quitting among healthcare workers. The findings reveal a statistically significant and positive influence of servant leadership on all dimensions of work engagement. These results align with previous studies, which have similarly affirmed the relationship between the observed variables [9,10,49].

Servant leadership positively impacts the vigor component of work engagement, as it fosters conditions that enhance employees’ intrinsic motivation and energy for work, as Zhou et al. [39] highlight. Vigor involves a high level of mental resilience, enthusiasm, and the persistence to exert effort and stay committed to tasks, even under challenging circumstances. Unlike hierarchical leadership approaches, servant leaders prioritize the needs of their employees, actively listen to them, offer support, and promote their growth and development. This leadership style helps build trust, security, and a sense of belonging among employees, reducing the fear of failure and alleviating stress [9]. In such an environment, employees perceive their work as meaningful, and their knowledge is valued, which motivates them to approach their tasks with greater energy. Furthermore, servant leaders recognize employees’ individual potential, reinforcing their sense of personal competence. When employees experience autonomy and emotional support, as demonstrated by Ozturk et al. [40], their engagement levels rise. Thus, servant leadership serves as an effective strategy for boosting vigor, as it addresses key psychological factors that help employees stay motivated and resilient in their work.

The study’s results highlight a positive influence of servant leadership on the dedication component of work engagement. Dedication encompasses commitment, enthusiasm, inspiration, and pride in performing work tasks. When leaders embrace a service-oriented approach, they show genuine concern for their employees’ needs and well-being, which enhances employees’ sense of value and strengthens their connection to the organization and its goals. As a result, employees begin to view their work as meaningful, which directly boosts their dedication. Additionally, servant leaders inspire employees through their own actions and the core values they promote, such as respect, ethics, and empathy [18,22]. When leaders actively foster a positive work culture, encourage open communication, and involve employees in decision-making, as Kaya and Karatepe [41] suggest, employee commitment is further enhanced. Therefore, servant leadership not only fosters a positive emotional relationship with work but also encourages sustained energy and effort towards achieving the organization’s long-term objectives.

The study confirms that servant leadership positively influences the absorption component of work engagement, which involves full immersion in tasks, losing track of time, and finding it difficult to interrupt work due to a high level of concentration and commitment. Servant leadership fosters a work environment where employees feel supported, safe, and motivated to fully engage with their tasks, as noted by Zhou et al. [39]. By addressing both personal and professional needs, servant leaders reduce stressors in the work environment that could hinder attention and concentration, as also highlighted in a similar study [9]. Moreover, servant leadership promotes autonomy, allowing employees to find the most effective ways to complete their tasks, which enhances creativity and innovation. When employees feel in control of their work, they experience deeper cognitive and emotional engagement. In a work environment built on mutual trust, employees are more likely to become “lost in their work,” motivated, and focused. Thus, servant leadership strengthens absorption by fostering internal motivation and minimizing distractions in the work environment.

The results of the multi-group analysis indicate that servant leadership positively affects all dimensions of work engagement across all observed subgroups, although the strength of this influence varies depending on gender, age, and years within the healthcare organization. Among women, a slightly stronger influence was observed across all three relationships, which aligns with the perspective of Bavik [19], who notes that societal expectations differ according to gender. As highlighted by Eva et al. [36], empathy, ethics, and support are central values of servant leadership and hold greater significance in groups that place importance on the relational and emotional aspects of this leadership style. This may explain why women exhibit higher levels of vigor, dedication, and absorption when they work in an environment that supports their needs and recognizes their contributions. Regarding age groups, the strongest effect of servant leadership was observed among the youngest employees (<40), particularly in relation to vigor and absorption. This may be associated with the need of younger employees for guidance and developmental opportunities, which serve as motivational factors enhancing engagement. The middle-aged group (41–50) also demonstrates a positive effect, although somewhat more moderate, while the oldest group (>51) shows the weakest effect, especially concerning dedication. These findings are consistent with Zhou et al. [39], who emphasize that younger employees often seek work-related support and recognition for their achievements, whereas older employees tend to develop greater autonomy and are less dependent on leadership style for fostering work commitment. A similar pattern emerges regarding years within a healthcare organization. The strongest effect of servant leadership is observed among employees with 10–20 years of service across all engagement dimensions. Employees in the early stages of their careers in healthcare organizations strive for professional development, making leadership support crucial in fostering energy, motivation, and full commitment to work, as emphasized also by Coetzer et al. [18] and Zhou et al. [39]. For the least experienced employees (<10 years), the effect is moderate, suggesting that they are still in the process of establishing work habits. Conversely, among the most experienced employees (>20 years of service), the effect of servant leadership is weakest, likely reflecting established behavioral patterns and a higher degree of professional independence. This finding partially aligns with Trastek et al. [43], who associate servant leadership with long-term organizational stability rather than with intense individual responsiveness among the most experienced employees.

This study offers a unique theoretical contribution, particularly in the context of healthcare organizations, where research on work engagement remains relatively underexplored [11,12], especially regarding the influence of servant leadership on healthcare workers’ engagement. Furthermore, this research provides significant theoretical insights by positioning the relationship between servant leadership and work engagement as a potential strategy to reduce or eliminate quiet quitting, a more recent and concerning phenomenon in the workforce. This study shows that servant leadership not only enhances work engagement but also mitigates quiet quitting, extending the relevance of social exchange theory [46,47]. When considering the specific context of the Republic of Serbia, this study holds additional value. To the best of the author’s knowledge, no similar studies have been conducted in Serbia, thus making the findings especially relevant and important for understanding work dynamics in developing countries. This adds substantial value to the scientific discourse by filling a gap in research within a developing country context.

In addition to the direct impact of servant leadership on work engagement, the results have broader implications for organizational phenomena such as bullying, inequality, and psychological safety. Servant leadership can significantly reduce the occurrence of bullying by fostering a climate of trust and compassion among employees [65], which is particularly relevant in healthcare organizations, where teamwork not only enhances the quality of care but also facilitates the development of positive interpersonal relationships. Previous research demonstrates that servant leadership strengthens the psychological safety of healthcare workers, which indirectly reduces burnout and promotes greater emotional and cognitive commitment among employees [66], in this study, specifically nurses. These findings align with the results obtained, which indicate that the influence of servant leadership is strongest among women and younger employees, who tend to seek greater support and opportunities for development. At the same time, recent research highlights that servant leadership can serve as a mechanism to mitigate structural inequalities, including gender disparities, by promoting equal development opportunities for all employees [67]. Therefore, by enhancing work engagement, servant leadership not only reduces the likelihood of quiet quitting but also indirectly contributes to the creation of a fairer work environment, minimizing negative outcomes such as bullying, inequality, and lack of psychological safety.

To translate our study findings into practice, healthcare organizations should institute targeted leadership development on servant skills, such as active listening, empowerment, humility, and ethical stewardship, using workshops, simulations, and mentoring, reinforced by 360° feedback aligned to servant-leadership behaviors. Although the selection of key managers in healthcare settings is not based on clear criteria and achieved performance in previous positions, it should explicitly weigh servant-leadership competencies in selection and promotion and link managerial incentives to team engagement. Educational institutions, especially those that develop study programs in the field of healthcare management, can integrate servant-leadership modules into curricula and promote short learning cycles on this topic. Policymakers and accrediting bodies can accelerate adoption by embedding leadership-for-engagement standards in accreditation and requiring proof of embedded leaders’ behavior as direct feedback from healthcare professionals.

The study has several limitations, which also provide directions for future research. Firstly, while the research assumes that the influence of servant leadership on work engagement could serve as a proactive strategy for reducing or eliminating quiet quitting, future studies should specifically measure and examine the quiet quitting phenomenon, as well as the role of servant leadership in addressing it. An interesting field for future research could be to investigate the intervening role of work engagement in this relationship, as well as the personal characteristics of the leader, organizational culture, or work-related outcomes. Additionally, increasing the sample size in future studies would lead to more precise and reliable results. This study was conducted in the Republic of Serbia, where market conditions and the context of healthcare organizations differ significantly from those in developed Western countries. A comparative analysis between different contexts could provide more robust conclusions. Robustness testing was performed on categorical variables such as gender, age, education, and years within organizations, but significant noninvariance was not detected. Future research should incorporate factors such as education, job position, and employment status (non-permanent employment vs. permanent employment) into multi-group analysis to provide a more comprehensive understanding of the research problem. Finally, the discriminant validity analysis showed that between the constructs of Empowerment and Humility the distinctiveness is threatened. Although conclusions about the positive effect of servant leadership on the work engagement relationship remain intact at the global construct level, this indicates the need for additional reconfiguration of the model through a facet-specific approach for Empowerment and Humility that integrates them into a composite indicator and re-estimates the model. Future work should refine Serbian item wordings (e.g., cognitive interviews), test measurement invariance across settings, and evaluate whether clearer wording increases distinctiveness between these facets.

## 6. Conclusions

This study demonstrates that servant leadership exerts a robust, positive effect on healthcare workers’ engagement—elevating vigor, dedication, and absorption. Accordingly, cultivating servant-leader behaviors in healthcare settings offers a concrete organizational lever for boosting engagement and, by extension, helping to mitigate quiet quitting. Based on the analysis of the influence of servant leadership on the dimensions of vigor, dedication, and absorption, the research confirmed that this leadership style has a statistically significant and positive impact on all three components of work engagement. Vigor is enhanced through the support and recognition of employee needs, which results in greater energy and resilience at work. Dedication is increased by creating a sense of meaning, belonging, and motivation, which servant leaders foster by valuing each employee’s contribution. Absorption is cultivated in an environment that servant leaders create, one that is free from distractions and promotes autonomy, allowing employees to focus deeply on their tasks. Overall, servant leadership demonstrates a significant effect on work engagement across all demographic groups, though the intensity varies. The stronger effect observed among women, younger employees, and those with medium tenure suggests that this leadership style is most impactful in the settings of greatest need for support, development, and recognition of individual capacities. Although the constructs of Standing back and Courage did not achieve the required validity, and two statements within the constructs of Empowerment and Stewardship did not meet reliability standards, the measurement scales otherwise demonstrated optimal values in the context of the Republic of Serbia. The positive relationship between servant leadership and work engagement was identified, suggesting that the servant leadership approach, which emphasizes the needs, development, and knowledge engagement of healthcare workers, can enhance work engagement. By fostering work engagement, servant leadership contributes to creating a work environment built on mutual trust, respect, and support, which may help reduce the occurrence of quiet quitting.

## Figures and Tables

**Table 1:** Respodent characteristics.

Category	Frequency	%
Gender		
Female	316	87.3
Male	46	12.7
Age		
<40	178	49.2
40–50	92	25.4
>51	92	25.4
Years within a healthcare organization		
<5	45	12.4
5–10	55	15.2
11–15	75	20.7
16–20	61	16.9
>20	126	34.8

Notes: N = 362.

**Table 2:** Measurement model and constructs.

Construct and Items		Loadings	VIF	α	Composite Reliability	AVE
SL: Servant Leadership						
EM: Empowerment				0.917	0.920	0.709
	EM01	0.778	3.123			
	EM02	0.884	2.921			
	EM03	0.862	2.138			
	EM04	0.777	3.152			
	EM05	0.875	2.848			
	EM06	0.868	1.905			
AC: Accountability				0.815	0.827	0.730
	AC01	0.806	1.882			
	AC02	0.885	4.038			
	AC03	0.870	1.904			
FO: Forgiveness				0.880	0.880	0.807
	FG01	0.901	4.655			
	FG02	0.914	4.540			
	FG03	0.879	3.914			
AU: Authenticity				0.887	0.889	0.747
	AU01	0.857	2.364			
	AU02	0.844	2.228			
	AU03	0.885	2.776			
	AU04	0.871	2.515			
HU: Humility				0.903	0.904	0.720
	HU01	0.809	2.530			
	HU02	0.839	2.274			
	HU03	0.852	3.976			
	HU04	0.868	2.693			
	HU05	0.873	2.707			
ST: Stewardship				0.905	0.905	0.913
	ST01	0.956	4.243			
	ST02	0.955	3.145			
Work Engagement						
VI: Vigor				0.800	0.816	0.628
	VI01	0.740	1.523			
	VI02	0.855	1.972			
	VI03	0.849	1.906			
	VI04	0.716	1.427			
DE: Dedication				0.741	0.774	0.660
	DE01	0.845	1.679			
	DE02	0.883	1.791			
	DE03	0.697	1.285			
AB: Absorption				0.764	0.786	0.586
	AB01	0.695	1.359			
	AB02	0.844	1.689			
	AB03	0.796	1.607			
	AB04	0.718	1.375			

**Table 3:** Discriminant validity.

Constructs	1	2	3	4	5	6
1. AC: Accountability	0.854					
2. AU: Authenticity	0.768	0.881				
3. EM: Empowerment	0.840	0.831	0.842			
4. FO: Forgiveness	0.826	0.840	0.836	0.923		
5. HU: Humility	0.817	0.824	0.887	0.858	0.858	
6. ST: Stewardship	0.776	0.803	0.794	0.785	0.773	0.955

**Table 4:** Structural model fit indices.

Construct	Stoner-Geisser Q^2^	R^2^	GOF	f^2^
Absorption	0.369	0.379	0.373	0.611
Dedication	0.332	0.341	0.336	0.518
Vigor	0.378	0.384	0.381	0.624
SRMR	0.062			

**Table 5:** Results of testing direct effects.

Relationship	Path Coefficient		*t*-Value	95% CIs (Bias Corrected)	Results
Formation of higher-order variables of Servant Leadership					
EM → SL		0.270 ***	44.316	[0.258, 0.282]	Supported
AC → SL		0.136 ***	30.451	[0.128, 0.145]	Supported
FO → SL		0.150 ***	32.766	[0.142, 0.161]	Supported
AU → SL		0.178 ***	32.812	[0.167, 0.188]	Supported
HU → SL		0.232 ***	40.378	[0.220, 0.243]	Supported
ST → SL		0.096 ***	28.607	[0.090, 0.103]	Supported
Servant Leadership effect on Work Engagement					
SL → VI		0.621 ***	16.098	[0.537, 0.687]	Supported
SL → DE		0.586 ***	13.991	[0.496, 0.660]	Supported
SL → AB		0.616 ***	16.370	[0.532, 0.682]	Supported

Notes: SL, Servant Leadership; EM, Empowerment; AC, Accountability; FO, Forgiveness; AU, Authenticity; HU, Humility; ST, Stewardship; VI, Vigor; DE, Dedication; AB: Absorption. *** *p* < 0.001.

**Table 6:** Robustness test: multigroup partial least squares path modeling.

Relationship	β	*t*	*p*	β	*t*	*p*	β	*t*	*p*	Invariant
Gender										
	Female	Female	Female	Male	Male	Male				
SL → VI	0.622	15.008	0.000	0.621	6.085	0.000				Yes
SL → DE	0.586	12.830	0.000	0.605	5.959	0.000				Yes
SL → AB	0.624	15.538	0.000	0.628	6.750	0.000				Yes
Age										
	<40	<40	<40	41–50	41–50	41–50	>51	>51	>51	
SL → VI	0.729	19.083	0.000	0.547	6.711	0.000	0.485	5.036	0.000	Yes
SL → DE	0.688	16.307	0.000	0.596	7.376	0.000	0.343	3.048	0.001	Yes
SL → AB	0.698	17.861	0.000	0.595	7.052	0.000	0.449	4.805	0.000	Yes
Years within a healthcare organization										
	<10	<10	<10	10–20	10–20	10–20	>20	>20	>20	
SL → VI	0.572	9.609	0.000	0.760	18.522	0.000	0.456	5.215	0.000	Yes
SL → DE	0.565	8.053	0.000	0.724	15.617	0.000	0.403	4.687	0.000	Yes
SL → AB	0.581	10.023	0.000	0.751	17.883	0.000	0.470	5.492	0.000	Yes

## Data Availability

The datasets generated and/or analyzed during the study are available from the corresponding author upon reasonable request.
